# Correlates of severity in a clinical staging model of schizophrenia: a cross-sectional study among 158 subjects

**DOI:** 10.1186/s12888-023-05144-6

**Published:** 2023-09-04

**Authors:** Fatima Hamieh, Souheil Hallit, Chadia Haddad, Sahar Obeid, Francois Kazour

**Affiliations:** 1 Faculty of Sciences, Lebanese University, Fanar, Lebanon; 2 School of Medicine and Medical Sciences, Holy Spirit University of Kaslik, P.O. Box 446, Jounieh, Lebanon; 3 Research Department, Psychiatric Hospital of the Cross, Jal Eddib, Lebanon; 4 Applied Science Research Center, Applied Science Private University, Amman, Jordan; 5 Institut National de Santé Publique, d’Épidémiologie Clinique et de Toxicologie-Liban (INSPECT-LB), Beirut, Lebanon; 6 School of Health Sciences, Modern University for Business and Science, Beirut, Lebanon; 7 School of Medicine, Lebanese American University, Byblos, Lebanon; 8 Social and Education Sciences Department, School of Arts and Sciences, Lebanese American University, Jbeil, Lebanon; 9 Department of Psychiatry, CHU Angers, Angers, France

**Keywords:** Staging, Schizophrenia, Psychosis, Mood, Antipsychotics

## Abstract

**Background:**

Clinical staging has been widely used to predict and optimize the treatment of medical disorders. Different models have been proposed to map the development, progression, and extension of psychiatric disorders over time, mainly for schizophrenia. The primary objective of this study was to classify patients with psychosis according to the McGorry staging model and compare factors between the different stages.

**Methods:**

This was a cross-sectional study, collecting data from 158 patients hospitalized for schizophrenia/psychosis. The survey included the Mini International Neuropsychiatric Interview (MINI), Positive and Negative Symptom Scale (PANSS), Montgomery-Asberg Depression Rating Scale (MADRS), Yong Mania Rating Scale (YMRS), Clinical Global Impression (CGI) scale, and the McGorry staging model.

**Results:**

Patients have been classified into three clinical stages: relapse of psychotic disorder (43%), multiple relapses (47.5%), and persistent and severe illness (9.5%). A higher mean duration of hospitalization, psychotic symptoms (PANSS total scale and subscales), chlorpromazine equivalent dose, and number of antipsychotic treatments were found among participants in Stage 4 as compared to the other groups. However, a significantly higher mean GAF scale was found among participants in stage 3b as compared to the other groups.

**Conclusion:**

Each stage in the McGorry staging model of schizophrenia is associated with well-defined clinical presentations, which help decide the appropriate treatment. Using such models in psychiatry can improve the diagnostic process and potential therapeutic interventions for patients suffering from mental disorders.

## Introduction

Schizophrenia is characterized by a wide range of symptoms, such as hallucinations, delusions, disorganized speech or behavior, and alteration in cognitive function [1]. This psychiatric disorder is disabling for the patients and their families because of its early onset and chronic nature [1]. Negative and cognitive symptoms, such as deficiencies in attention, working memory, or executive function, frequently combine to cause disability. Additionally, positive symptoms including suspicion, hallucinations and delusions might lead to relapse [1].

The best way to define disorders that progress in a complicated fashion is through stages, which will not only identify a specific point in the disease’s progression but also the best course of treatment at that stage [2]. This strategy has proven to be quite effective for organizing the treatment in oncology throughout time [2]. It has been hypothesized that staging models can be crucial for treatment planning for a complicated disorder like schizophrenia [2]. Various conceptual staging models have been put forth. These models sought to categorize the clinical stages (prodromal, initial episode, acute phase, remission and relapse, chronic phase and residual symptoms) of schizophrenia development.

Among the proposed classifications, some included a premorbid phase or increased risk without frank psychotic symptoms. Fava and Kellner presented a staging model for schizophrenia as follows: Stages 1 and 2 are for prodromal phases and acute episodes respectively, Stage 3 is for residual symptoms, Stage 4 is for subchronic symptoms (lasting between 6 months and 2 years) but more than 6 months), and Stage 5 is for chronic symptoms lasting more than 2 years) [3].

Lieberman proposed that schizophrenia consists of three pathophysiologic phases divided in four stages [3]. Stage 1 or the premorbid phase, also known as the neurodevelopmental phase, starts in early adolescence and is characterized by mild cognitive and social abnormalities. The neuroplastic phase, (Stages 2 and 3) includes the prodromal phase followed by the presence of mild psychotic symptoms. Stage 3 is the presence of full-blown psychosis. Finally, the neuro progressive phase is characterized by chronic or residual psychotic symptoms with significant negative and cognitive impairment [4].

The chronology of psychosis progression is the primary emphasis of Singh and coll. in their staging model. The prodromal phase (stage 1), is split into two phases: a phase of unease (P1) and a phase of non-diagnostic symptoms (P2). Stage 2 begins with the first positive symptoms of psychosis, such as delusions and hallucinations. Stage 3 is a transitional phase characterized by worsening symptoms, followed by Stage 4 that confirms the diagnosis of schizophrenia [5].

According to Agius et al., the three stages of the development of schizophrenia are the prodrome (stage 1), the initial episode (stage 2), and the chronic phase (stage 3). This author also supports that there exists a premorbid phase prior to the prodromal phase, despite the fact that it was not accounted for as a separate stage [2].

McGorry and coll., proposed one of the most elaborate staging models of schizophrenia [3]. Their theoretical model begins with stage 0, when the patient has no symptoms but an elevated risk for psychosis. Then, stage 1 is split into two sub-stages: 1a, which includes mild and non-specific symptoms, and 1b, which includes moderate/subthreshold symptoms. Stage 2 consists of the first episode of psychosis, while stage 3 is divided into three substages: incomplete remission (3a), first relapse (3b), and recurring relapses (3c). Stage 4 indicates a severe and enduring disease. Patients with schizophrenia spectrum disorder as well as other mood disorders including depression or bipolar illness can also benefit from this staging model. The model presented by McGorry et al. also included information on possible therapeutic modalities [6].

As for Cosci et al., they present a four-stage model, including the prodromes (Stage 1), the acute manifestation (stage 2), the residual symptoms (stage 3), and the chronic phase (stage 4) [3].

Finally, Fountoulakis et al. (2019) proposed a clinical staging model using the the 5-factor model (5 categories of symptoms related to schizophrenia: positive, negative, affective, cognitive and hostility symptoms) and the Positive and Negative Syndrome Scale (PANSS) [7] This model identified 4 major clinical stages of schizophrenia after studying a population of stabilized patients diagnosed with schizophrenia with varying ages [8].

So far, no studies have evaluated the correlation between staging models and clinical presentation and severity among patients with chronic schizophrenia and long-term hospitalizations in psychiatric settings. Among these classification models, the McGorry staging model may be one of the most developed classifications and has the potential to match the clinical stage to the intervention. In this model, the clinical stages are well defined, and the target populations for recruitment are mentioned. Moreover, the sub-stages of McGorry’s classification make this model easily adaptable to the various clinical presentations of schizophrenia, in both outpatient and inpatient settings.

In this context, the formulated research question is the following: Can we use a staging model in a sample of patients diagnosed with schizophrenia, to understand better the clinical presentation, the target treatment, and the functional outcome of this population? So far, only the Diagnostic and Statistical Manual of Mental Disorders (DSM) classification is used in this context and cannot be a comparator for this approach since it cannot determine the outcome, the clinical severity, or the target treatment. Therefore, the present study aims to classify a population of Lebanese in-patients with schizophrenia according to the McGorry staging model and compare factors between the different stages.

## Methods

### Study design and participants

This cross-sectional study, evaluating long-term hospitalized patients with psychosis, was conducted at the Psychiatric Hospital of the Cross, Lebanon, one of the largest psychiatric hospitals in the Middle East, between August and October 2021. The inclusion criteria were: diagnosis of schizophrenia according to the Diagnostic and Statistical Manual of Mental Disorders, 5th edition (DSM-5) criteria, age between 18 and 65 years, and hospital stays for more than one year [9]. Exclusion criteria were the presence of somatic or physical conditions preventing the patients from participating in the interviews (acute somatic condition, deterioration in general condition or extreme fatigue due to a physical condition, preventing the person from taking part in an assessment interview), dementia, intellectual disability, cognitive impairment (according to the Mini-Mental State Exam), substance use disorders (except nicotine and caffeine), and refusal to answer the questions. 223 patients were screened during the study period; among 87 female patients, 26 patients were excluded due to age limit, 1 patient due to intellectual disability, and 8 patients refused to participate. Among 136 male patients, 30 patients were excluded due to the age limit. The final sample consisted of 158 respondents (52 females and 106 males).

### Questionnaire

The questionnaire was in Arabic, with an average response time of 50 min. Data was collected by a trained person through a personal interview. The first section of the questionnaire inquired about socio-demographic variables (age, gender, marital status, and educational attainment). The second section included questions about medical history, duration and onset of illness, duration of hospitalization in psychiatry, number of admissions to the psychiatric hospital, family history of psychiatric condition, and chronic medical condition. Ongoing treatments including antipsychotics, antidepressants, mood stabilizers, lithium, anxiolytics, and hypnotics were also recorded. Chlorpromazine equivalent doses were calculated according to the minimum effective dose method [10]. Moreover, different scales were included in the questionnaire as follows:

The Mini International Neuropsychiatric Interview (MINI) [11] is a structured diagnostic interview, developed jointly by psychiatrists and clinicians in the United States and Europe, for the Diagnostic and Statistical Manual of Mental Disorders, 4th edition (DSM-4) and International Classification of Diseases, 10th revision (ICD-10) psychiatric disorders. With an administration time of approximately 15–20 min, it was designed to meet the need for a short but accurate structured psychiatric interview for multicenter clinical trials and epidemiology studies and to be used as a first step in outcome tracking in clinical settings. The MINI comprises modules for 17 psychiatric diagnoses. Questions are phrased to allow only “yes” or “no” answers. One point is scored every time a patient answers “yes” to a question.

The Positive and Negative Syndrome Scale (PANSS) [7], validated in Arabic [12], is a medical scale used for measuring positive (7 items), negative (7 items), and general psychopathology (16 items) symptom severity of patients with schizophrenia. The scale is a “gold standard” used for most assessments of psychotic behavioral disorders. Scoring of PANSS ranges from 7 to 49 for each of the positive and negative subscales, and from 16 to 112 for the general psychopathology subscale. Higher scores reflect more severe symptoms of psychosis.

The Montgomery–Åsberg Depression Rating Scale (MADRS) [13], validated in Arabic [14], is a ten-item diagnostic questionnaire used to measure the severity of depressive episodes in patients with mood disorders. Each item has a severity scale from 0 to 6 with higher scores reflecting more severe symptoms. Scoring of the MADRS scale ranges from 0 to 60. Scores of 0–6 indicate an absence of symptoms, 7–19 represent mild depression, 20–34 Moderate, 35–60 indicate severe depression.

The Young Mania Rating Scale (YMRS) [15] is an eleven-item multiple choice diagnostic questionnaire used to measure the presence and severity of mania and associated symptoms. Each item is composed of five explicitly defined levels of severity. Scoring of the YMRS ranges from 0 to 60. Higher scores indicate severe manic symptoms.

The Global Assessment of Functioning (GAF) Scale [16] is a numeric scale used by clinicians and physicians to rate the social, occupational, and psychological functioning of an individual. GAF scores are divided into numerical categories ranging from 1 to 100. Each level is broken down into groups of 10 and starting at 100. Higher scores indicate greater levels of functioning. The most favorable mental health functioning is represented by scores that range from 91 to 100. Those with minor psychological problems are rated in the 71 to 90 range of functioning. Severe mental health concerns fall in the 21 to 30 range. Ratings that range from 1 to 10 are reserved for those who are incapable of meeting minimal standards of personal care.

The CGI Scale of Clinical Global Impression [17] consists of three different global measures: Severity of illness (CGI-S), Global Improvement (CGI-I), Efficacy index (CGI-I). Each component of the CGI scale is rated separately, without yielding a global score. Items 1 and 2 are rated on a 7-point scale; item 3 is rated from 0 to 4. Though widely used in clinical psycho-pharmaceutical trials, the CGI Scale brings benefits to all levels of psychiatric treatment.

Severity of Illness (CGI-S): The severity of illness subscale is designed to acquaint the patient’s severity of symptoms with those of other people experiencing the same mental ailment. The CGI-S rates this severity on a 1–7 scale, with higher scores reflecting more severe illness.

### Clinical staging

For clinical staging, we used the three clinical indicators used by McGorry [6] [18]: The severity of symptoms, using the PANSS total score [7]; Recurrence or relapses based on the number of lifetime psychotic episodes that were taken from the patients’ medical files; and the global functioning, using the GAF score [16]. These five clinical stages ranged from favorable functioning and no symptoms (stage 2) to unremitted illness and poor functioning (stage 4). According to the characteristics of our study, which included only patients after at least a first psychotic episode, stages 0, 1a, and 1b were not part of the study, given that they describe patients at risk of psychosis.

### Statistical analysis

The SPSS software version 25 was used to perform data analysis. The quantitative variables were considered as normally distributed as verified by the visual inspection of the histogram, while the skewness and kurtosis were within |1.96 [19] except for the total PANSS, general psychopathology PANSS subscale, and MADRS scores. For these scales, the median and interquartile range were reported. For the remaining quantitative scales, the means and standard deviation were reported, whereas categorical variables were expressed as absolute frequencies and percentages. The Chi-square and Fisher exact tests were used to test the association between categorical variables, whereas the ANOVA test was used to compare three or more means. *P* < .05 was considered significant.

## Results

### Sociodemographic characteristics

Table 1 shows the demographic and other characteristics of patients with schizophrenia. The mean age of the patients was 52.34 ± 8.64 years, with 67.1% males. The majority (91.1%) were single, with a low education level (complementary level and below: 71.6%). Only 25.3% have a family history of psychiatric illness and 30.4% have a history of medical illness. The mean age of onset of symptoms was 25.00 ± 7.73 years, the duration of psychiatric illness was 27.01 ± 10.62, the duration of hospitalization in years was 15.11 ± 8.74, and the number of hospitalizations was 6.15 ± 6.10.

**Table 1:** Sociodemographic and other characteristics of the participants (N = 158)

Variable	N (%)
Gender	
Male	106 (67.1%)
Female	52 (32.9%)
**Marital status**	
Single/divorced/widowed	144 (91.1%)
Married	14 (8.9%)
**Education level**	
Illiterate	8 (5.1%)
Primary	36 (22.8%)
Complementary	69 (43.7%)
Secondary	32 (20.3%)
University	13 (8.2%)
**Family history of psychiatric illness**	
Yes	40 (25.3%)
No	118 (74.7%)
**History of medical illness**	
Yes	48 (30.4%)
No	110 (69.6%)
	**Mean ± SD**
**Age**	52.34 ± 8.64
**Age of onset of symptoms (years)**	25.00 ± 7.73
**Duration of psychiatric illness (years)**	27.01 ± 10.62
**Duration of hospitalization (years)**	15.11 ± 8.74

### Description of the participants’ clinical characteristics

Participants’ clinical characteristics are described in Table 2. According to the clinical staging for psychosis, 43.0% have recurrence or relapse of psychotic (Stage 3b), 47.5% have multiple relapses (Stage 3c) and 9.5% have severe, persistent, or unremitting illness (Stage 4). The majority of patients have moderate to severe illness (70.9%). Considering that all participants are inpatients presenting with psychotic symptoms for at least one year (minimal duration of hospitalization for inclusion), the results show that none of the participants were classified in stages 0, 1, or 2, corresponding to increased risk, mild symptoms, and first psychotic episode respectively.

**Table 2:** Description of participants clinical characteristics

	Frequency (%)
Clinical staging for psychotic	
Stage 3b :Recurrence or relapse of psychotic	68 (43.0%)
Stage 3c :Multiple relapses	75 (47.5%)
Stage 4 :Severe, persistent, or unremitting illness	15 (9.5%)
**Clinical Global Impressions (CGI)**	
Mildly ill	46 (29.1%)
Moderately ill	58 (36.7%)
Markedly ill	39 (24.7%)
Severely ill	15 (9.5%)
	**Mean ± SD**
**Total PANSS scale**	59.68 ± 16.56 (Median = 56; IQR = 21)
Positive PANSS subscale	16.94 ± 5.56
Negative PANSS subscale	15.68 ± 6.52
General psychopathology PANSS subscale	27.05 ± 7.37 (Median = 25; IQR = 8)
**Depression (MADRS scale)**	2.47 ± 3.85 (Median = 0.001; IQR = 4)
**Manic symptoms (YMRS scale)**	4.80 ± 3.27
**Global Assessment of Functioning (GAF scale)**	36.83 ± 7.30
**Chlorpromazine equivalent dose**	1179.69 ± 1024.12
**Number of antipsychotic treatment**	1.84 ± 0.84

The mean total PANSS score was 59.68 ± 16.56, the mean depression score (MADRS scale) was 2.47 ± 3.85 and the mean manic symptoms score (YMRS scale) was 4.80 ± 3.27. The mean chlorpromazine equivalent dose was 1179.69 ± 1024.12 mg and the mean number of antipsychotic treatments was 1.84 ± 0.84.

### Comparison of factors between the different clinical stages

The results showed that a significantly higher proportion of participants belonging to stage 3b as compared to the other stages were single (*p* = .046) and had a family history of psychiatric illness (*p* = .014). The type of treatment used by the participants did not show any significant association with the clinical staging (*p* > .05 for all). A higher mean duration of hospitalization (*p* = .001), psychotic symptoms (PANSS total scale and subscales) (*p* < .001 for all), chlorpromazine equivalent dose (*p* < .001), and number of antipsychotic treatments (*p* = .012) were found among participants in Stage 4 as compared to the other groups. However, a significantly higher mean GAF scale was found among participants in stage 3b as compared to the other groups (*p* < .001).

### Association between Clinical Staging, PANSS, and GAF scores

**Fig. 1:**
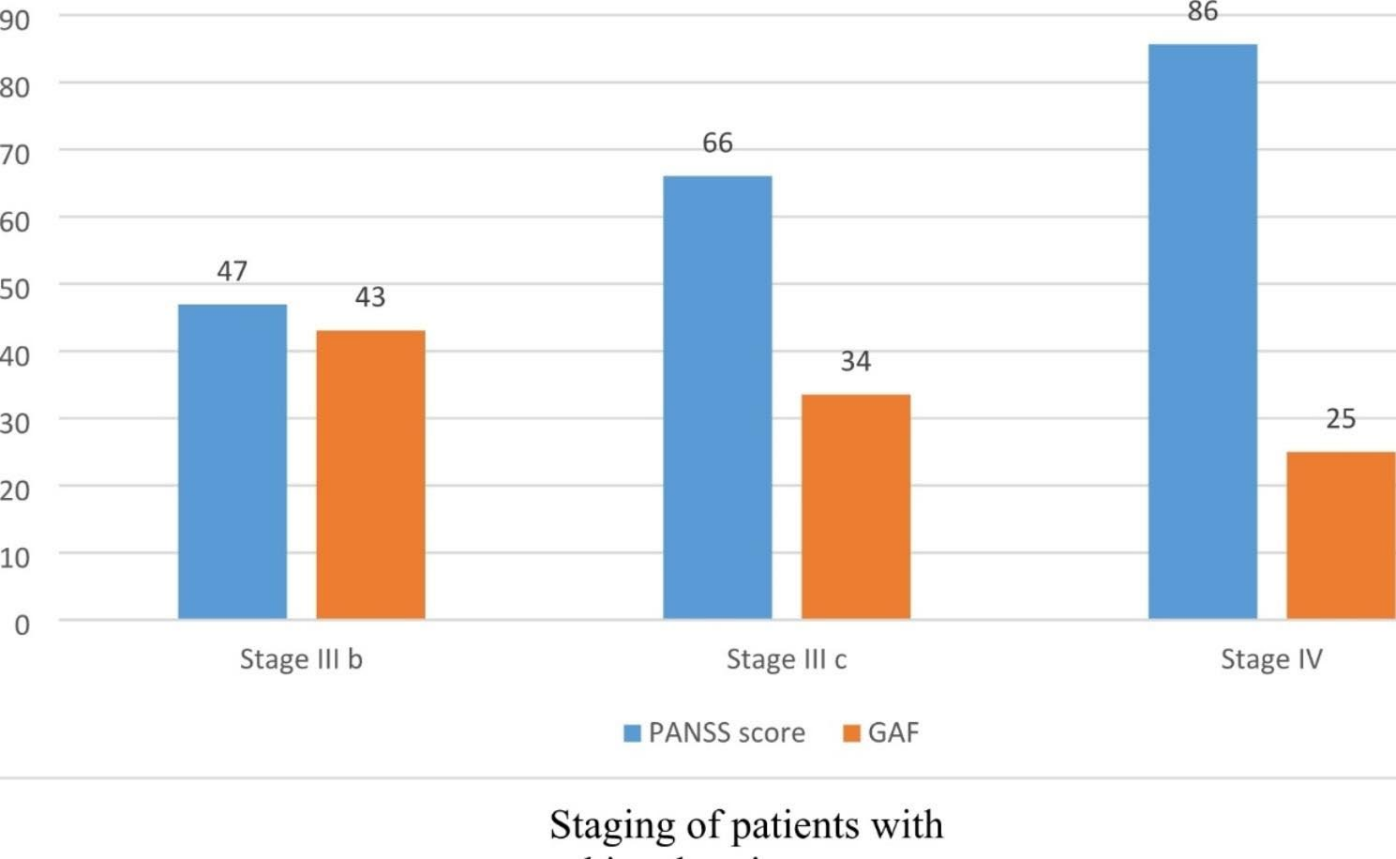
Comparison of clinical symptoms (PANSS scale) and Global Assessment of Functioning between the clinical stages of patients with schizophrenia

Patients were classified into three clinical stages, defined as Stage 3b (n = 68), Stage 3c (n = 75), and Stage 4 (n = 15). The results show a significant progressive increase in PANSS scores, and a decrease in GAF scores between Stages 3b, 3c, and 4, confirming the deterioration of patients’ clinical presentation between different stages. Stage 3b corresponds to patients with recurrence or relapse of psychotic (mean PANSS score 46.93 ± 6.76) and moderate impairment in functioning (mean GAF score 43.08 ± 3.9). In Stage 3c, patients have multiple relapses (mean PANSS score 66.04 ± 12.34) and severe impairment in functioning (mean GAF score 33.53 ± 4.84). Stage 4 represents the most advanced stage of the illness, corresponding to patients who have extremely high levels of symptomatology (PANSS score 85.67 ± 17.97) and highly impaired global functioning (mean GAF score 25.00 ± 0.01) (Table 3; Fig. 1).

**Table 3:** Comparison of factors between the different clinical stages

	Stage 3b	Stage 3c	Stage 4	*p*
	Frequency (%)	Frequency (%)	Frequency (%)	
Gender				
Male	41 (60.3%)	55 (73.3%)	10 (66.7%)	0.253
Female	27 (39.7%)	20 (26.7%)	5 (33.3%)	
**Marital status**				
Single/divorced/widowed	66 (97.1%)	66 (88.0%)	12 (80.0%)	**0.046**
Married	2 (2.9%)	9 (12.0%)	3 (20.0%)	
**Education level**				
Illiterate	3 (4.4%)	5 (6.7%)	0 (0%)	0.061
Primary	9 (13.2%)	21 (28.0%)	6 (40.0%)	
Complementary	28 (41.2%)	34 (45.3%)	7 (46.7%)	
Secondary	18 (26.5%)	12 (16.0%)	2 (13.3%)	
University	10 (14.7%)	3 (4.0%)	0 (0%)	
**Family history of psychiatric illness**				
Yes	24 (35.3%)	11 (14.7%)	5 (33.3%)	**0.014**
No	44 (64.7%)	64 (85.3%)	10 (66.7%)	
**History of medical illness**				
Yes	19 (27.9%)	23 (30.7%)	6 (40.0%)	0.654
No	49 (72.1%)	52 (69.3%)	9 (60.0%)	
**Type of treatment**				
Atypical Antipsychotics	33 (48.5%)	25 (33.3%)	3 (26.7%)	0.103
Typical Antipsychotics	52 (76.5%)	67 (89.3%)	13 (86.7%)	0.110
Benzodiazepines use	25 (36.8%)	30 (40.0%)	4 (26.7%)	0.617
Anti-epileptic	26 (38.2%)	42 (56.0%)	7 (46.7%)	0.104
Anti-cholinergic	49 (72.1%)	62 (82.7%)	14 (93.3%)	0.108
Anti-depressant - SSRI	5 (7.4%)	8 (10.7%)	0 (0.0%)	0.367
Anti-depressant TCA	9 (13.2%)	5 (6.7%)	1 (6.7%)	0.378
Mood stabilizer	13 (19.1%)	8 (10.7%)	3 (20.0%)	0.321
	**Mean ± SD**	**Mean ± SD**	**Mean ± SD**	
**Age**	51.62 ± 8.70	52.93 ± 8.46	52.67 ± 9.64	0.657
**Age of onset of symptoms in years**	26.17 ± 7.48	24.69 ± 8.17	21.20 ± 5.18	0.069
**Duration of psychiatric illness in years**	25.00 ± 11.13	28.02 ± 9.94	31.06 ± 10.30	0.069
**Duration of hospitalization in years**	12.29 ± 7.43	16.77 ± 9.28	19.60 ± 7.94	**0.001**
**Number of hospitalizations**	6.53 ± 8.09	5.48 ± 3.92	7.80 ± 4.09	0.324
**Total PANSS scale**	46.93 ± 6.76	66.04 ± 12.34	85.67 ± 17.97	**< 0.001**
Positive PANSS subscale	12.47 ± 2.64	19.35 ± 4.19	25.20 ± 4.39	**< 0.001**
Negative PANSS subscale	11.59 ± 3.95	17.87 ± 5.74	23.33 ± 7.66	**< 0.001**
General psychopathology PANSS subscale	22.87 ± 3.01	28.83 ± 6.29	37.13 ± 12.06	**< 0.001**
**Depression (MADRS scale)**	2.97 ± 3.63	2.00 ± 3.83	2.53 ± 4.86	0.325
**Manic symptoms (YMRS scale)**	4.19 ± 3.13	5.08 ± 3.18	6.13 ± 3.96	0.067
**Global Assessment of Functioning (GAF scale)**	43.08 ± 3.96	33.53 ± 4.84	25.00 ± 0.01	**< 0.001**
**Chlorpromazine equivalent dose**	848.03 ± 908.69	1354.33 ± 1029.89	1810.0 ± 1039.43	**< 0.001**
**Number of antipsychotic treatment**	1.63 ± 0.82	1.97 ± 0.83	2.20 ± 0.77	**0.012**

Numbers in bold indicate significant *p* values

## Discussion

Patients in our study belonged to stages 3b (43%), 3c (47.5%), and 4 (9.5%) only. In our study, higher positive, negative, and general psychopathology PANSS subscales scores, a higher number of antipsychotics, and a higher mean chlorpromazine equivalent dose were significantly associated with severe illness (Stage 4 compared to the other groups). These results are consistent with previous reports that suggest that there is a 25% increase in the PANSS total score before relapse [20]. Concerning the chlorpromazine equivalent dose, this result is consistent with the report that intensive antipsychotic dosage has great importance in the treatment of chronic schizophrenia [21]. However, a significantly higher mean GAF scale was found among participants in stage 3b as compared to the other groups, which is consistent with the McGorry staging model that shows a higher GAF score in stage 1b (< 70) than in stage 2 (GAF 30–50) [22].

The staging model would gain specificity if one or more quantifiable biological markers could be identified [23]. Several biomarkers reflecting possible causal mechanisms and/or consequences of the pathophysiology are candidates for integration into the clinical staging model of psychiatric illnesses [23]. Electroencephalography (EEG) can be used to measure the most important brain function impairments in the psychosis spectrum and severe mood disorders [23]. In clinical psychiatry, this could involve not only a cross-sectional biological definition but also a wider biopsychosocial definition of extent or progression [6]. Other motor indications, and neurocognitive disturbances could be included. Non-invasive biological markers, such as changes in brain volume can be detected using Magnetic Resonance Imaging (MRI). Genetic variables like Catechol-O-Methyl transferase (COMT) and serotonin-transporter gene, and other endocrine markers may be displayed to reflect progression or greater severity of the disorder may eventually be included. For biological phenomena, such as hippocampal atrophy in individuals with depression or schizophrenia, this may be related to the duration of untreated illness. A clinical staging model could then be used to determine which biological markers could ultimately be useful in treatment selection and prognosis [22]. The model could be further tailored for use in psychiatry, by including social factors, such as social isolation or vocational failure, which typically flows from poorly or mistreated treated illness. In any case, a person who presents for initial treatment with a great deal of collateral personal and social damage is less likely to respond to interventions (i.e. be more treatment resistant at that point whether primary or secondary) and hence more likely to have a worse prognosis [22].

### Clinical implications

Each stage in the McGorry staging model of schizophrenia is associated with well-defined clinical presentations. Staging system help in deciding appropriate treatment. The concept of a staging approach to the treatment of schizophrenia is gaining prominence [8, 24, 25]. Clinical staging is widely used in different medical specialties. Using such models in psychiatry can improve the diagnostic process and potential therapeutic interventions for patients suffering from mental disorders. If staging was applied at a large scale for psychiatric disorders such as schizophrenia, we would be able to assess treatment efficacy according to their ability to prevent progression from earlier to later stages. The use of a standardized model of staging would ensure that treatments that are offered earlier are effective, safe, acceptable, and affordable [2, 6].

### Limitations

For the limitations, we can mention the issue of sufficient awareness and training in phenomenological informed assessment of psychopathology [24]. A second related issue is that of inter-rater reliability. One significant challenge in connection with earlier pre-psychotic illness stages is that clinical ultra-high-risk patients may be quite heterogeneous concerning the pathophysiology and pathogenesis of psychotic disorders. Another challenge is associated with the transition from clinical to pathophysiological stage definitions [26]. For example, to date, it has been difficult to demonstrate robust and replicable relationships between brain abnormalities in schizophrenia patients and the severity of their clinical or neurocognitive impairments. In this study, we did not find stages 1, 2, and 3a, which is considered a limitation. The data was collected at a specific point in time. The follow-up of the patients was not evaluated. Data was collected from one hospital in Lebanon. Finally, rehabilitation and psychotherapeutic approaches may have had an impact on the prognosis of psychosis, hence on the clinical stage classification. In our study, all included subjects are considered as long-term inpatients (hospitalization for at least one year), and benefited from non-specific supportive group therapy, along with medical and psychiatric care. This may be a limitation, even though no specific individual psychotherapy or rehabilitation program was implemented for specific participants.

## Conclusion

The Staging model focuses primarily on dividing the course of the disorder into recognizable stages based on seriousness, development, and symptom characteristics to better predict prognosis. On the clinical side, defining discrete stages creates a framework for the evolution of interventions oriented toward prevention. In this study, we were able to classify 158 patients hospitalized for schizophrenia according to the McGorry staging model. This classification must aid clinicians select treatments that are particularly relevant to each stage. When considering the application of staging models to psychiatric disorders, applying such strategies to patients at earlier stages where intervention is more efficient to limit the progression of the illness, which is not the case in our study. Further studies must consider the indicative biological and endophenotypic markers, social, and protective factors for better classification.

## Data Availability

The datasets generated and/or analyzed during the current study are not publicly available due to restrictions from the ethics committee but are available from the corresponding author on reasonable request.

## References

[1] Patel KR, Cherian J, Gohil K, Atkinson D (2014). Schizophrenia: overview and treatment options. Pharm Ther.

[2] Agius M, Goh C, Ulhaq S, McGorry P (2010). The staging model in schizophrenia, and its clinical implications. Psychiatr Danub.

[3] Cosci F, Fava GA (2013). Staging of mental disorders: systematic review. Psychother Psychosom.

[4] Lieberman JA, Perkins D, Belger A, Chakos M, Jarskog F, Boteva K (2001). The early stages of schizophrenia: speculations on pathogenesis, pathophysiology, and therapeutic approaches. Biol Psychiatry.

[5] Singh SP, Cooper JE, Fisher HL, Tarrant CJ, Lloyd T, Banjo J (2005). Determining the chronology and components of psychosis onset: the Nottingham Onset schedule (NOS). Schizophr Res.

[6] McGorry PD, Nelson B, Goldstone S, Yung AR (2010). Clinical staging: a heuristic and practical strategy for New Research and Better Health and Social Outcomes for psychotic and related Mood Disorders. Can J Psychiatry.

[7] Kay SR, Fiszbein A, Opler LA (1987). The positive and negative syndrome scale (PANSS) for schizophrenia. Schizophr Bull.

[8] Fountoulakis KN, Dragioti E, Theofilidis AT, Wikilund T, Atmatzidis X, Nimatoudis I (2019). Staging of Schizophrenia with the Use of PANSS: An International Multi-Center Study. Int J Neuropsychopharmacol.

[9] DSM Library [Internet]. [cited 2022 Dec 9]. Diagnostic and Statistical Manual of Mental Disorders. Available from: https://dsm.psychiatryonline.org/doi/book/10.1176/appi.books.9780890425787.

[10] Leucht S. Dose equivalents for second-generation antipsychotics: the minimum effective dose method. Schizophr Bull. 2014;40(2). 10.1093/schbul/sbu001 PMC3932104 24493852

[11] Sheehan DV, Lecrubier Y, Sheehan KH, Amorim P, Janavs J, Weiller E (1998). The mini-international neuropsychiatric interview (M.I.N.I.): the development and validation of a structured diagnostic psychiatric interview for DSM-IV and ICD-10. J Clin Psychiatry.

[12] Hallit S, Obeid S, Haddad C, Kazour F, Kazour GR (2017). Validation of the Arabic Version of the PANSS scale among lebanese schizophrenic patients. J Psychopathol.

[13] Montgomery SA, Asberg M (1979). A new depression scale designed to be sensitive to change. Br J Psychiatry J Ment Sci.

[14] Hallit S, Obeid S, El Hage W, Kazour F (2019). Validation of the arabic version of the MADRS scale among lebanese patients with depression. L’Encéphale.

[15] Young RC, Biggs JT, Ziegler VE, Meyer DA (1978). A rating scale for mania: reliability, validity and sensitivity. Br J Psychiatry J Ment Sci.

[16] Aas IM (2011). Guidelines for rating Global Assessment of Functioning (GAF). Ann Gen Psychiatry.

[17] Busner J, Targum SD (2007). The Clinical Global Impressions Scale. Psychiatry Edgmont.

[18] Godin O, Fond G, Bulzacka E, Schürhoff F, Boyer L, Myrtille A (2019). Validation and refinement of the clinical staging model in a french cohort of outpatient with schizophrenia (FACE-SZ). Prog Neuropsychopharmacol Biol Psychiatry.

[19] Kim HY (2013). Statistical notes for clinical researchers: assessing normal distribution (2) using skewness and kurtosis. Restor Dent Endod.

[20] Wang D, Gopal S, Baker S, Narayan VA (2018). Trajectories and changes in individual items of positive and negative syndrome scale among schizophrenia patients prior to impending relapse. NPJ Schizophr.

[21] OLLENDORFF RH (1960). High dosage chlorpromazine therapy in acute and chronic schizophrenia. Am J Psychiatry.

[22] McGorry PD, Hickie IB, Yung AR, Pantelis C, Jackson HJ (2006). Clinical staging of psychiatric disorders: a heuristic framework for choosing earlier, safer and more effective interventions. Aust N Z J Psychiatry.

[23] Lavoie S, Polari AR, Goldstone S, Nelson B, McGorry PD (2019). Staging model in psychiatry: review of the evolution of electroencephalography abnormalities in major psychiatric disorders. Early Interv Psychiatry.

[24] Nelson B, McGorry PD, Fernandez AV (2021). Integrating clinical staging and phenomenological psychopathology to add depth, nuance, and utility to clinical phenotyping: a heuristic challenge. Lancet Psychiatry.

[25] Borbála Dombi Z, Barabássy Á, Sebe B, Laszlovszky I, Németh G. Clinical Staging in Schizophrenia Spectrum Disorders. In: Fukao K, editor. Psychosis - Phenomenology, Psychopathology and Pathophysiology [Internet]. IntechOpen; 2022 [cited 2023 Mar 14]. Available from: https://www.intechopen.com/chapters/76958.

[26] Mathalon DH (2011). Challenges associated with application of clinical staging models to psychotic disorders. Biol Psychiatry.

